# The Strength of the Corticospinal Tract Not the Reticulospinal Tract Determines Upper-Limb Impairment Level and Capacity for Skill-Acquisition in the Sub-Acute Post-Stroke Period

**DOI:** 10.1177/15459683211028243

**Published:** 2021-07-04

**Authors:** Ulrike Hammerbeck, Sarah F. Tyson, Prawin Samraj, Kristen Hollands, John W. Krakauer, John Rothwell

**Affiliations:** 1 Geoffrey Jefferson Brain Research Centre, 158986 Manchester Academic Health Science Centre, Faculty of Biology, Medicine and Healthy, 5292 University of Manchester, Manchester, UK; 2 Department of Health Professions, Faculty of Health, Psychology and Social Care, 5289 Manchester Metropolitan University, Manchester, UK; 3 Department of Medical Physics, Northern Care Alliance NHS Trust, Salford, UK; 4 Department of Health Sciences, 105168 University of Salford, Salford, UK; 5 Departments of Neurology, Neuroscience and Physical Medicine & Rehabilitation, 1500 The John Hopkins University School of Medicine, Baltimore, MD, USA; 6 The Santa Fe Institute, Santa Fe, NM, USA; 7 Institute of Neurology, University College London, London, UK

**Keywords:** stroke, upper limb, motor impairment, skill learning, corticospinal tract, reticulospinal tract

## Abstract

*Background*. Upper-limb impairment in patients with *chronic* stroke appears to be partly attributable to an upregulated reticulospinal tract (RST). Here, we assessed whether the impact of corticospinal (CST) and RST connectivity on motor impairment and skill-acquisition differs in *sub-acute* stroke, using transcranial magnetic stimulation (TMS)–based proxy measures. *Methods*. Thirty-eight stroke survivors were randomized to either reach training 3-6 weeks post-stroke (plus usual care) or usual care only. At 3, 6 and 12 weeks post-stroke, we measured ipsilesional and contralesional cortical connectivity (surrogates for CST and RST connectivity, respectively) to weak pre-activated triceps and deltoid muscles with single pulse TMS, accuracy of planar reaching movements, muscle strength (Motricity Index) and synergies (Fugl-Meyer upper-limb score). *Results*. Strength and presence of synergies were associated with ipsilesional (CST) connectivity to the paretic upper-limb at 3 and 12 weeks. Training led to planar reaching skill beyond that expected from spontaneous recovery and occurred for both weak and strong ipsilesional tract integrity. Reaching ability, presence of synergies, skill-acquisition and strength were not affected by either the presence or absence of contralesional (RST) connectivity. *Conclusion*. The degree of ipsilesional CST connectivity is the main determinant of proximal dexterity, upper-limb strength and synergy expression in sub-acute stroke. In contrast, there is no evidence for enhanced contralesional RST connectivity contributing to any of these components of impairment. In the sub-acute post-stroke period, the balance of activity between CST and RST may matter more for the paretic phenotype than RST upregulation per se.

## Introduction

Motor impairment after stroke is closely associated with ipsilesional corticospinal tract (CST) damage.^1-4^ In addition, recent data suggest that arm flexor synergies, finger enslaving on the paretic side and mirror movements on the non-paretic hand after stroke are all attributable to an increased influence of the reticulospinal tract (RST) after damage to the CST.^5-11^ Studies in primates have shown that 6 months after a lesion in the pyramidal tract,^12^ there is upregulation of the RST. In patients with *chronic* stroke, the incidence of contralesional connectivity to the ipsilateral paretic limb is increased, particularly in patients with moderate to severe paresis,^13,14^ suggesting a similar upregulation of RST activity during recovery.^15^ An unanswered question is the impact of this RST upregulation after the initial plegic stage^3^; does it contribute to, or impede recovery, or is it an epiphenomenon of recovery, neither good nor bad.^7^ Furthermore, it is unclear whether unwanted muscle synergies result from actual upregulation of pre-existing cortico-reticulospinal descending pathways or can be attributed instead to a relative imbalance between them (in the absence of upregulation) and the CST.^6^

Using transcranial magnetic stimulation (TMS), we sought to determine the degree of ipsilesional and contralesional cortical connectivity to paretic arm muscles in a group of patients with moderate to severe stroke in the early sub-acute period. TMS of the human motor cortex in one hemisphere can evoke responses in ipsilateral muscles with characteristics compatible with activation of oligosynaptic cortico-bulbospinal pathways,^16^ most likely representing cortico-reticulo-spinal connection.^13,14,17-19^ This provides an indirect method of assessing the excitability of the RST in stroke survivors.^11,20-22^ We further investigated the effect of these two forms of connectivity on strength, synergies, planar reaching accuracy and capacity for skill-acquisition. We examined inputs to proximal muscles involved in planar reaching movements since these are thought to receive greater reticulospinal inputs than distal arm muscles.^16,23^

## Materials and Methods

The parallel (1:1 allocation) randomized controlled study was approved by the North West, Greater Manchester West Research Ethics Committee 15/NW/0703, registered as ISRCTN 81668376. Informed consent was obtained from each participant according to the Declaration of Helsinki.

### Participants

**Figure 1.**
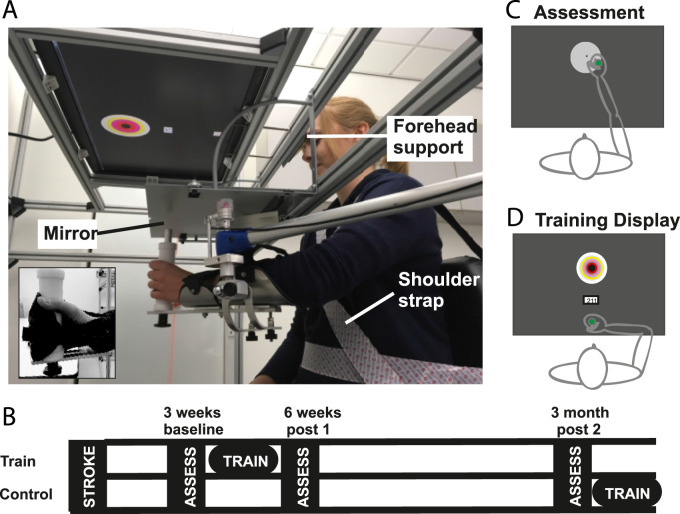
(A) Experimental set-up for reaching training and reaching accuracy assessment. Inset demonstrates use of glove to secure hand to handle. (B) Study flow diagram. (C) Visual display during reaching accuracy assessment and (D) during training sessions.

Clinical Research Network practitioners screened acute admissions to three Northwest England stroke units for stroke survivors with arm weakness and established consent for contact by the research team. All participants met the following inclusion criteria: (1) Sub-acute stroke survivor (in the previous 3 weeks) with (2) upper-limb weakness (≤4 Medical Research Council Scale) of either triceps or anterior deltoid muscles, (3) ability to perform ≥15 cm weight supported reach in robotic manipulandum (Figure 1A) and (4) engaging in therapy sessions. We excluded individuals with (1) history of previous stroke or other concomitant neurological or musculoskeletal disease, (2) contraindication to TMS,^24^ (3) cerebellar stroke, (4) proximal upper limb hypertonus ≥3 on modified Ashworth scale (MAS), (5) severe sensory impairment (<6/12 Fugl-Meyer sensory scale), (6) shoulder pain ≥3/10 on self-rated continuous visual analogue scale, (7) new self-reported uncorrected visual impairment, (8) hemi-spatial neglect established by the Star Cancellation Task and (9) cognitive and language impairment impeding co-operation in study protocol.

Participants were randomized (using www.rando.la by a researcher (KH) not involved in data acquisition), stratified to age (<65 years) and Fugl-Meyer score (FMS) (<50), to either an active intervention (high repetition reach training and usual care) or control group (usual care only). Usual care comprised physiotherapy and occupational therapy either as inpatient or with the early supported discharge team. All participants attended for an assessment at 3 weeks (baseline), 6 weeks (post1) and 12 weeks (post2) after stroke (Figure 1B).

### Apparatus and Stimuli

Reaching was performed using a mobile arm support (SAEBO MAS, SAEBO Inc., Charlotte, NC) (Figure 1A) fitted with rotary encoders (Bourns Inc., AMS22S5A1BHBFL336 with a resolution of 4096 steps/revolution) to detect displacement. The device was modelled into the Robotics Module in LabVIEW 2016 for calibration and kinematic calculations. Angles obtained from rotary encoders were processed using the ‘Forward Kinematics’ Virtual Instrument to obtain the handle’s location in relation to the starting position. All kinematic data were sampled at 100 Hz. Compensatory movements were prevented by a forehead support, shoulder strap and backrest support (Figure 1A). Participants held a custom-made handle, using a custom-made glove if necessary (Figure 1A inset). A forearm support eliminated gravity. Vision of the hand was occluded by a mirror, which displayed feedback. Feedback comprised a 2 × 2 cm starting box, a green cursor (0.5 cm diameter) representing the handle position and a circular 10 cm diameter target, located 20 cm from the start box at 90°.^25,26^ To test accuracy, the target was displayed as a white disc (Figure 1C) and during training as a bullseye with 1 cm spaced concentric circles (Figure 1D). When movement was initiated and tangential velocity exceeded 3 cm/s, the green cursor disappeared. It only reappeared to display feedback of the end position for 1 second, after the movement stopped, and velocity dropped below 5 cm/s. Cursor feedback during movement was removed to prevent corrective movements, which could compensate for reduced skill and thereby make differences in skill harder to detect.

Reaching was performed at individualized (1) self-selected, (2) slow and (3) fast movement speeds to vary task difficulty and maintain interest. Each individual’s movement speed was determined as described previously.^25^ In brief, after task familiarization (15 repetitions with and without visual feedback of hand position), participants were encouraged to reach as quickly as possible in a third set of 15 movements. The 80th percentile or fourth shortest movement time from these fast movements was used to set the limit for the individual’s fast movement time. The slow movement time was limited to movements 200 ms slower than this with a maximum movement time of 2000 ms.^25,26^ No movement speed limit was imposed during self-selected movement speed blocks.

### Intervention

The training was designed to approach 400 reaches for sufficient task practice to promote motor learning.^27^ As the protocol was performed in a sub-acute stroke population who received concurrent rehabilitation,^28^ it was acknowledged that daily sessions were likely to be too difficult to fit in from both a logistical and fatigue perspective. The training group received 6 training sessions, performed 2-3 times/week, between baseline and the post1 assessments (Figure 1B). The control group was offered training after their post2 assessment. Training sessions lasted 1-1½ hours, aiming to perform up to 420 accurate reaches to the bullseye target (Figure 1D) (7 blocks of 60 repetitions; three blocks at self-selected (SS) and two each at slow and fast movement speed). The average number of reaching movements/session was 377 (±8.6 SD) reaches. Accurate movements were rewarded with five points for terminating in the bullseye (<1 cm error) and incremental reduction to one point in the outer ring (4-5 cm error) with a maximum of 300 points (60 × 5 points) per block. Accumulative points/block was displayed on the screen and a beep indicated when the movement was within the speed limit and the target area receiving at least 1 point. Movements that ended outside the target area and/or did not fall within the required movement limit were awarded zero points. Breaks between blocks were a minimum of 30 seconds, but individuals were permitted longer breaks as needed to avoid fatigue.

### Outcome Measures

#### Clinical Measures

Trained clinical research practitioners blinded to group allocation performed all clinical tests. Upper-limb impairment and synergy expression were measured with the FMS (/66) and sensory subscales, upper-limb strength with the Motricity Index (MI) (/99) and elbow flexor hypertonus with modified Ashworth scale.^29^ The summed NIHSS score and upper-limb sub-score documented at acute admission measured stroke severity.

#### Reaching Accuracy

To assess reaching accuracy, participants performed 6 blocks (2 at self-selected speed, 2 slow and 2 fast) of 20 planar reaching movements.^25,26^ The initial block was always at self-selected speed and the other blocks were randomly interspersed. Reaching accuracy was defined as the unsigned absolute error between reaching termination and the target centre and calculated as the mean error on all reaches of the assessment day. This was also expressed as the spread of the endpoint location around this constant error (variable error).^25,30^

#### Corticospinal Integrity

EMG activity was recorded with self-adhesive Ag/AgCl electrodes (Skintact®) using a muscle belly montage for triceps brachii (lateral head) and anterior deltoid as per SENIAM EMG recording recommendations.^31-33^ EMG signals were amplified (1000×) and band-pass filtered (fourth order 30 Hz-500 Hz) with a custom-built data acquisition device (2015-28 16-channel EMG). The signals were digitized by sampling at 2 kHz using a custom-built laboratory interface (developed in LabVIEW 2016) and stored on a laboratory computer for display and off-line data analysis with custom written LabVIEW software scripts.

Single pulse TMS was delivered using a 70-mm figure-of-eight shaped TMS coil and a Magstim 200 magnetic stimulator (Magstim Company, Whitland, Dyfed, UK). The coil was placed tangentially over the scalp over M1 with the handle pointing postero-laterally at 45° to the sagittal plane inducing a posterior–anterior current in the brain. To achieve muscle pre-activation, individuals were instructed to perform a bimanual phasic forward reaching movement against a weak elastic band.^16,23^ To ensure consistent pre-activation, we triggered the TMS pulse when triceps activity reached 20% maximal voluntary contraction (MVC).^34^ The MVC of the affected triceps was established while seated in a supportive chair by the greatest EMG excursion during three maximal-effort reaching movements against resistance.

The active motor threshold (aMT) for the unaffected triceps muscle was established at baseline when stimulating the contralesional hemisphere. The motor hotspot and aMT for paretic triceps and deltoid muscle were recorded when stimulating the affected (ipsilesional hotspot) and unaffected (contralesional) hemisphere.^35^ If no MEPs were detected in the affected upper limb, the mirror symmetrical hotspot location for the unaffected triceps was used.^20^ A train of 20 stimulations was delivered at 120% aMT or 100% maximum stimulator output (MSO) if no aMT could be established, to the ipsilesional and contralesional hotspot, while recording MEPs for the affected upper limb. MEP recordings were overlaid for visualization^17^ and individual traces investigated by a custom written LabVIEW script to ensure a consistent response in more than 50% of traces.^17^ A response was classified as an increase in background EMG around the expected interval that exceeded ongoing EMG by at least 1 SD for a period of 5 ms.^16^ We classified individuals into no, only ipsilesional, only contralesional or both ipsi- and contralesional connectivity.

We investigated the influence of ipsilesional connectivity strength on baseline performance and change due to training. Ipsilesional connectivity strength was expressed as a percentage of the aMT of the affected, compared to the unaffected side: (1) Affected aMT < 125% unaffected (strong connectivity), (2) affected aMT > 125% unaffected (weak connectivity) or (3) no MEP observed (no connectivity).

Contralesional connectivity was established by applying TMS to the unaffected hemisphere. Connectivity was defined as present when stimulation up to 100% of stimulator output elicited a consistent MEP in either triceps, deltoid or both muscle groups. We investigated how performance differed between individuals with only ipsilesional connectivity and those with contralesional and ipsilesional connectivity.

#### Motor Threshold, MEP Latency and Amplitude

We investigated whether the aMT (%MSO when stimulating ipsilesional or contralesional hemisphere) and response latency (in triceps and deltoid) changed in the affected upper-limb in individuals with MEPs at baseline.

### Data Analysis

The data were analysed using custom written MATLAB® (Mathworks) routines and IBM SPSS software on an intention to treat basis (*P* <= .05, distribution normality confirmed by the Kolmogorov–Smirnov test).

The association between ipsilesional (3) and contralesional (2) connectivity and baseline impairment (reaching accuracy, MI and FMSs) were assessed using one-way ANOVA. Group differences were investigated by repeated measure ANOVAs Time(2) × Group(2) × Connectivity(2-3) for change from baseline to post2 for connectivity in either the ipsilesional (3) and contralesional pathways (2).The effect of training was assessed by two separate rmANOVA Time(2) × Group(2), baseline to post1 and baseline to post2. Post hoc paired t-test assessed performance change with Bonferroni correction for the two time points (significant *P* <= .025).

Changes to aMT from baseline to post2 in the contralesional and ipsilesional pathways were assessed by repeated measure ANOVA Pathway(2) × Time(2) × Group(2) and differences of affected and unaffected ipsilesional aMT at baseline by Student’s t-test. The changes in MEP latency, amplitude and normalized amplitude were analysed in both triceps and deltoid muscle with repeated muscle ANOVA Time(2) × Muscle(2) × Group(2) in both pathways.

## Results

Thirty-eight participants were recruited (19 active and 17 control), two withdrew before randomization. Thirty-two (16 in each group) were analysed at post1; one withdrew without giving a reason, one did not like the TMS, for one their circumstances changed, and another was lost to follow-up (Table 1). Twenty-nine participants were included in the analysis for post2.

**Table 1.** CONSORT Flow Diagram of Study Enrolment and Retention.

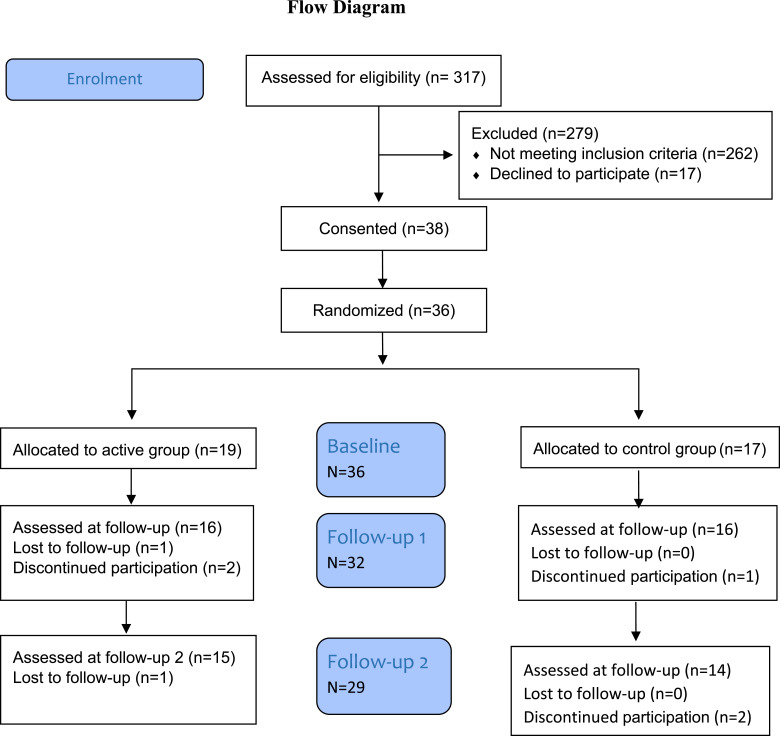

Participants’ baseline demographics (Table 2) indicate similar ages with more male participants (63% vs 47%), slightly lower FMS (30.2 vs 32.6) and MI scores (48.8 vs 54.3) in the training group than the control group. Both groups had significant fatigue (Fatigue Severity Scale 37.4 vs 38.7), with average to moderate impairment (NIHSS score 11 vs 9) and varying hypertonus on the modified Ashworth scale.

**Table 2.** Demographics of Study Participants at Baseline (Mean and SD Except When Other Measure Stated).

Baseline	Baseline/Post2	Training, n = 19	Control, n = 17	*P*
Age (median/range in years)	Baseline	61 (range 28-94)	62 (range 42-86)	*P* = .92
Gender (%)				
Male	Baseline	63	47	*P* = .11
Female		27	53	
Affected arm (%)				
Left	Baseline	56	61	*P* = .74
Right		44	39	
NIHSS all (/42) (median/range)	Baseline	11 (range 3-25)	9 (range 3-23)	Mann Whitney; *P* = .68
NIHSS arm (/4) (%)	Baseline	0 = 0%; 1 = 5%, 2 = 11%, 3 = 16%, 4 = 68%	0 = 0%; 1 = 12%, 2 = 24%, 3 = 29%, 4 = 35%	Mann Whitney; *P* = .08
Sensation Fugl-Meyer subset (/12)	Baseline	9.6 (±3.1)	11.1 (±1.4)	*P* = .12
Fugl-Meyer UL (/66)	Baseline	30.2 (±16.1)	32.6 (±18.1)	*P* = .69
	Post2	44.3 (±17.3)	42.5 (±17.3)	*P* = .78
UL Motricity Index (/99)	Baseline	48.8 (±23.5)	54.3 (±23.8)	*P* = .52
	Post2	70.1 (±21.5)	64.3 (±24.3)	*P* = .51
Hypertonus (MAS) prevalence of score	Baseline	0 = 33%; 1 = 17%; 1+ = 44%; 2 = 6%	0 = 61%; 1 = 11%; 1+ = 6%; 2 = 22%	Mann Whitney; *P* = .56
	Post2	0 = 50%; 1 = 7%; 1+ = 29%; 2 = 7%; 3 = 7%	0 = 29%; 1 = 29%; 1+ = 7%; 2 = 14%; 3 = 7%	Mann Whitney; *P* = 1.0
Fatigue Severity Scale (/63)	Baseline	37.4 (±18.7)	38.7 (±19.9)	*P* = .86
	Post	35.9 (±17.9)	41.1 (±19.0)	*P* = .47

### Contralesional Connectivity Was Not Increased Above Normal Levels 3 or 12 Weeks After Stroke

**Figure 2.**
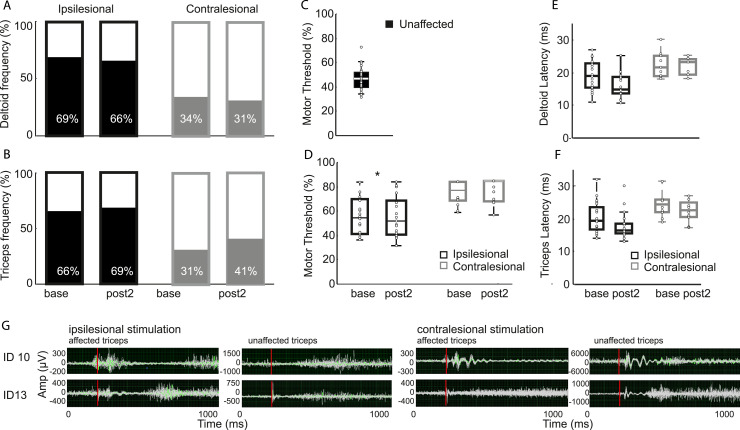
Frequency of ipsilesional and contralesional connectivity obtained from the paretic upper limb observed at baseline and 12-week follow-up in (A) deltoid and (B) triceps muscle. Active motor threshold (aMT) for (C) unaffected contralateral MEPs when stimulating the unaffected hemisphere and (D) MEPs seen in the weak affected arm for ipsilesional and contralesional responses at baseline and post2. Changes in MEP latency for (E) deltoid MEPs and (F) triceps MEPs from baseline to follow-up at 12 weeks. (G) Twenty overlaid triceps EMG traces for two subjects (ID10 and ID13) who both have MEPs in the affected upper limb when the lesioned hemisphere is stimulated. ID10 also has MEPs in the affected upper limb when the contralesional hemisphere is stimulated in contrast to ID13, who does not demonstrate this.

We assessed MEPs in the weak upper-limb, elicited by stimulating the ipsilesional and contralesional hemisphere at baseline and post2 with TMS (Figures 2A and 2B). At baseline, we observed ipsilesional connectivity in deltoid in 69% (23/32) and in triceps in 66% (22) participants; contralesional MEPs were seen in deltoid in 34% (11/32) and in triceps in 31% (10) participants. In seven individuals, we observed contralesional responses in both triceps and deltoid. At post2, an ipsilesional MEP was elicited in deltoid in 66% (19/29) and in triceps in 69% (20) of participants; a contralesional MEP was observed in deltoid in 34% (9/29) and in triceps in 41% (12) of participants. The fact that ipsilesional stimulation could only elicit MEPs in two-thirds of the sample is indicative of their stroke severity, whereas the contralesional hemisphere was within normal limits for healthy adults.^36^

When investigating MEP characteristics, the aMT (Figures 2C and 2D) for contralateral MEPs was greater when stimulating the affected cortex than the unaffected cortex (56.6 ± 16.7 vs 42.7 ± 8.0, t_(24)_ = 4.87, *P* < .001). The aMT, to elicit MEPs in the affected arm, was greater when stimulating the contralesional than ipsilesional hemisphere (mean = 74.2 ± 9.8 vs 54.2 ± 9.8, t_(13)_ = 5.8, *P* < .001). A two-way rmANOVA revealed a significant effect of Time (F_(1,10)_ = 26.65, *P* < .001) and Pathway (F_(1,10)_ = 38.18, *P* < .001) without an interaction. The aMT for the connection from the ipsilesional cortex (t_(19)_ = 3.94, *P* = .001) and contralesional cortex (t_(10)_ = 3.21, *P* = .009) decreased over time.

Similarly, ipsilesional response latency (Figure 2E) decreased (Time F_(2,28)_ = 10.54, *P* <= .001) in both triceps ((t_(17)_ = 3.84, *P* = .001) and deltoid muscle(t_(16)_ = 3.63, *P* = .002). However, there was no change in contralesional latency (Time F_(2,6)_ = .683, *P* = .540) (Figure 2F) for either muscle. The MEP response latency in the unaffected triceps was 15.1 ms (±1.4 ms) after stimulation of the contralesional hemisphere.

The MEP amplitude was smaller in triceps than deltoid (Muscle: F_(1,14)_ = 7.071, *P* = .019, base amplitude triceps vs deltoid: t_(18)_ = −2.76, *P* = .013) but did not change in either muscle or between groups for either the ipsilesional (Time F_(2,28)_ = 1.078, *P* = .354) or contralesional pathway (Time F_(2,4)_ = 2.30, *P* = .253). (Triceps: ipsilesional base = 213 μV ± 376, post2 = 278 μV ± 421; contralesional base = 131 μV ± 139, post2 = 338 μV ± 338. Deltoid: ipsilesional base = 440 μV ± 566, post2 = 527 μV ± 492; contralesional base = 247 μV ± 203, post2 = 271 μV ± 103.) Similarly, there was no difference in the MEP normalized to the pre-activation EMG in the ipsilesional (F_(2,28)_ = .25, *P* = .784) or contralesional (F_(2,4)_ = .981, *P* = .45) pathway or between groups.

In summary, we found that MEPs in the affected upper-limb were diminished when stimulating the ipsilesional hemisphere in our study population. In a third of our population, we could not elicit a MEP and when we could, the motor threshold was higher, the latency longer and the amplitude smaller in comparison to MEPs from the unlesioned hemisphere to the unaffected triceps and deltoid muscles. For contralesional connectivity, the prevalence of observing a connection was not increased and only the motor threshold reduced over time.

### Ipsilesional Not Contralesional Connectivity Measures Were Associated With Baseline Motor Performance, Recovery of Strength and Reduction in Synergies

We investigated whether connection strength was related to the FMS and the MI at baseline and over time. The control and training groups are combined for the analysis as there was no difference in impairment at 12 week follow-up (rmANOVA Time(2) × Group(2) no interaction or effect of Group: FMS F_(1,27)_ = .006, *P* = .937, Motricity F_(1,25)_ = .113, *P* = .740.)

**Figure 3.**
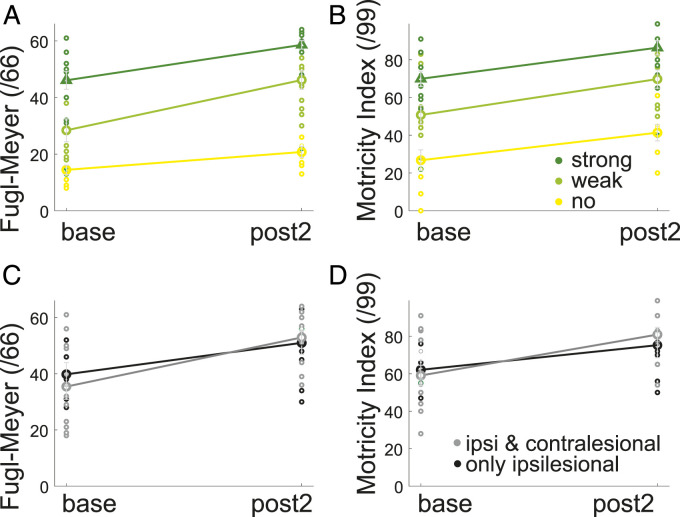
Association between ipsilesional corticospinal connectivity strength (no = yellow, weak = light green and strong = dark green) and impairment at baseline and at post2, for the (A) Fugl-Meyer Upper Limb Score and (B) MI. Association between having only ipsilesional (black) or ipsilesional and additional contralesional connectivity (grey) and impairment at baseline and post2 for (C) FMS and the D) MI.

We found an association between ipsilesional cortical connectivity and the FMS (Figure 3A) (main effect of connectivity F_(2,26)_ = 29.7, *P* < .001) and that baseline FMS were significantly different between individuals with strong (47.4 ± 10.7, n = 6), weak (27.6 ± 14.8, n = 12) and absent connections (14.3 ± 5.3, n = 7) (onewayANOVA F(2,31) = 17.86, *P* < .001). The FMS changed over time without an interaction (effect of Time F_(1,26)_ = 42.3, *P* < .001).

The same effect of ipsilesional connectivity was seen for the MI (Figure 3B) (main effect of Motricity F_(2,24)_ = 22.6, *P* < .001). The MI score was significantly different between individuals with strong (71.2 ± 15.1, n = 5), weak (46.4 ± 16.7, n = 11) and absent connections (25.6 ± 18.1, n = 7) at baseline (onewayANOVA F(2,31) = 15.87, *P* < .001) and similarly changed over time (effect of Time F_(1,24)_ = 39.46, *P* < .001) without an interaction.

For the group with ipsilesional connectivity (Figures 3C and 3D), there was no difference between individuals with only ipsilesional connectivity (n = 11) and individuals with additional contralesional connectivity (n = 13) for either the FMS (Main effect of Connectivity F_(1,22)_ = 2.3, *P* = .147 or the MI (Main effect of Connectivity F_(1,20)_ = 3.9 *P* = .061) nor an interaction.

In summary, baseline impairment was associated with the strength (or absence) of connectivity from the ipsilesional hemisphere to the paretic limb and was unrelated to degree of contralesional connectivity. All clinical measures improved between 3 and 12 weeks but did not interact with the degree of ipsi- or contralesional connectivity.

### Acquisition of Skilled Reaching Was the Same for Weak and Strong Ipsilesional Connectivity and Unaffected by the Presence or Absence of Contralesional Connectivity

**Figure 4.**
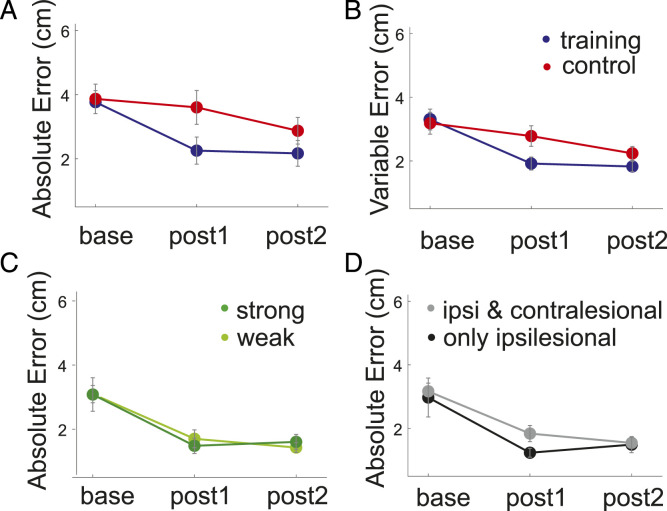
(A) Absolute and (B) variable reaching endpoint error at baseline, post1 and post2 for the training (blue) and control (red) group. (C) Association between weak (light green) and strong (dark green) ipsilesional corticospinal connectivity strength and changes in absolute reaching endpoint error from baseline to post2. (D) Association between having only ipsilesional (black) or ipsilesional and additional contralesional connectivity (grey) and reaching accuracy at baseline and post2.

To investigate how differences in connectivity strength affected training-induced skill-acquisition, we excluded the sub-group of patients without any connectivity because their baseline skill was much lower, which greatly complicates comparisons of change.^37^ Reaching accuracy increased over time in both groups (Figure 4A). The two-way rmANOVA main effect of Time (F_(1,29)_ = 14 101, *P* = .001) was however significantly greater in the training (mean = 1.38 cm) than control group (mean = .26 cm; t_(29)_ = −2.42, *P* = .022). The improvement was still evident at post2 (Time F_(1,28)_ = 5.76 *P* = .023) but did not differ between groups. Improvement over time was also seen in the reaching endpoint’s variable error (Figure 4B) (main effect of Time: F_(1,29)_ = 30.11, *P* < .001) and differed between groups Time × Group interaction (F_(1,29)_ = 7.72, *P* = .009). The accuracy improvement was not at the cost of movement duration (baseline mean training pre = 957.4 ms ± 116.7 ms, post2 = 968.6 ms ± 148.74 ms; control pre = 959.2 ± 149.1 ms, post2 = 922.6 ± 128.8). A two-way rmANOVA with Time and reaching speed as main factors showed no main effect of Time (F_(1,30)_ = .299, *P* = .589) and no Time × Group interaction (F_(1,30)_ = 1.062, *P* = .311).

Reaching skill improved over time (effect of Time F_(1,21)_ = 23.16, *P* < .001) but did not differ between individuals with weak (n = 11) or strong (n = 6) CST connectivity (Figure 4C) (weak = 3.09 ± .7, strong = 3.08 ± 1.3, no main effect of Connectivity F_(1,21)_ = 1.005, *P* = .328, Time × Connectivity Interaction F_(1,21)_ = .049, *P* = .826).

Baseline reaching accuracy was no better in patients with contralesional cortex connectivity to paretic muscles in addition to an ipsilesional connection (n = 12 vs n = 11) (mean = 2.9 ± 1.4 vs 3.2 ± .7; t_(10)_ = −.31, *P* = .76) (Figure 4D). These results show that in the presence of similar initial performance, varying degrees of CST integrity can lead to the same degree of skill change. In addition, changes in descending connectivity from the unaffected hemisphere plays no role in this kind of skill-acquisition in individuals with matched baseline ability and some CST connectivity.

## Discussion

We sought to investigate the relationship between TMS-derived connectivity measures from the ipsilesional and contralesional hemispheres and levels of motor impairment and capacity for skill-acquisition in early sub-acute stroke. The ipsi- and contralesional measures^20^ were taken as proxies for CST and RST integrity, respectively.^21^

At 3 weeks post-stroke, we observed reduced ipsilesional connectivity but no evidence for upregulation of contralesional connectivity. In addition, there was no increased contralesional connectivity at 12 weeks. Weakness and the presence of synergies at baseline were inversely correlated with the strength of ipsilesional connections but, in contrast to findings in chronic stroke,^7,8^ were not related to either the presence or absence of contralesional connectivity. We found that skill-acquisition in a planar reaching task, after matching for initial performance, did not depend either on the strength of ipsilesional connectivity or on the state of contralesional connectivity.

### Measuring CST Connectivity With TMS

We employed robust methodology^13,14,17^ to establish presence or absence of connectivity and changes in MEP characteristics.^4,13,20,38^ Although we observed a reduction in the active motor threshold and latency of ipsilesional responses over the 12-week period for both groups (both indications of stronger corticospinal connectivity), we did not see an increase in MEP size in the unaffected limb for either group. Our study protocol emphasized presence/absence of response rather than absolute amplitude which could make detection of change harder. However, peak-to-peak MEP amplitude is not the best measure of response size for the polyphasic nature of EMG responses typical following stroke. A better measure might have included response duration as well as amplitude, but the presence of background contraction and the small size of the responses made this impractical. Comparison of responses at different time points during the period of spontaneous recovery would require robust normalization methods to account for differences in surface electrode placement and pre-activation.^39^ In addition, different pre-activation levels can affect MEP onset detection when using a threshold decision rule.^39^ We therefore limited the interpretation of these responses to the presence or absence of connectivity.^14^

### Measuring Presumed RST Connectivity With TMS

TMS has been widely used to probe presumed cortico-reticulo-spinal projections from the contralesional hemisphere to the affected upper-limb.^16,20^ These responses are multifaceted and have significant inter-stimulus variability, which makes response quantification difficult.^16^ We used TMS output up to 100% MSO but because of the use of a figure-of-eight coil and the laterality of the proximal arm response hotspots (∼3 cm lateral from midline), we are confident that observed contralesional responses were not due to current spread to the ipsilesional hemisphere.^4,40,41^

### The Relationship of CST Integrity to Initial Impairment and Subsequent Recovery

We observed reduced ipsilesional connectivity, consistent with a reduction in CST integrity post-stroke, which was related to baseline impairment: the more excitable the CST was to TMS, the less impairment was observed. Over time, impairment improved and CST connections were strengthened (demonstrated by reduced latencies and motor thresholds^20^). We conclude that CST connections provide the basis for recovery from impairment, which in turn allows for training-related functional improvements.^1^

### The Relationship of a Detectable Contralesional RST to Impairment and Recovery

In the presence of damage to the CST, alternative pathways from the contralesional hemisphere, specifically the RST provide a potential additional connection and therapeutic target for recovery.^12,15,42,43^ In chronic stroke, RST connectivity is increased^13,14^ and has been associated with greater CST damage and resultant motor impairment.^8^ However, whether this additional connectivity contributes to impairment or alternatively aids recovery^3^ may depend on the muscle group examined. The RST is important for trunk and proximal movements, innervating motoneurons over multiple levels.^44^ An increase in connectivity from the contralesional cortex to ipsilateral paretic shoulder flexors and trunk muscles has been observed in chronic stroke survivors.^14,45^ In non-human primates, increased RST connectivity to flexors has been observed after a pyramidal lesion.^12^ This is likely due to preferential ipsilateral innervation from reticular formation neurons to flexor muscles whereas contralateral innervation is more prevalent to extensor muscles.^46^ However, in chronic stroke, increased contralesional connectivity to the affected triceps muscle has also been observed.^13,14^ This may be explained by the multiple cortical areas in both hemispheres that reticulospinal neurons are innervated by.^42^

Due to its multi-level innervation, it has been proposed that RST upregulation is detrimental to normal movement by favouring upper-limb flexor synergies and reducing movement fractionation.^7,15^ However, we did not see upregulation of contralesional connectivity either early or at 12 weeks follow-up. Therefore, at least in the first 3 months after stroke, the presence of synergies cannot be attributed to upregulation of the RST from the contralesional hemisphere. We propose instead that at this early stage in recovery, synergies are the result of an imbalance in drive between the damaged CST drive and the preserved RST. Good recovery in this stage would entail upregulation of CST projections to reverse the imbalance and restore CST dominance. An example of this could be the improved performance in reaching function observed after training with increasing abduction load.^47^ Poor recovery results if CST upregulation cannot occur, which may lead to increased RST connectivity at a later stage, as seen in the primate at 6 months post-stroke^12^ and in chronic stroke survivors.^7,8^

### The Relationship Between the Strength of CST Integrity and the Ability to Acquire a Skill

When matching for initial impairment, patients with a detectable CST could increase their planar reaching skill, in agreement with previous work demonstrating that stroke survivors can improve their movement skill with training,^25,48^ as observed in healthy individuals.^49^ Interestingly, skill improvement was the same in individuals with strong or weak CST connectivity. That is, when initial performance was the same, learning was also the same. Initial performance was likely matched because the planar reaching task was fully weight-supported, allowing the capacity of the residual CST to be fully expressed.^50,51^ Thus, in this gravity-supported reaching task involving proximal muscles, it does not seem that CST integrity is an independent predictor of skill-acquisition, beyond its relation to initial performance. This is perhaps not unexpected as skill-acquisition is more likely attributable to cortical changes,^52^ with the resultant optimized commands transmitted via the CST, which may only need some lower-bound or threshold level of connectivity.

### Limitations

Our study cohort of stroke survivors with clear weakness early after stroke with varied degree of recovery complicates data analysis. We investigated the relationship between recovery and connectivity in muscles involved in planar reaching movements; future research should establish if these findings generalize to dexterous finger movements.

Despite stimulating at intensities of up to 100% of stimulator output, several participants were MEP negative for both ipsilesional and/or contralesional connectivity, reducing group sizes and making correlation analysis difficult. This highlights the severity of damage caused by the stroke in some participants as observed in previous studies.^13,20^ Gaining insights into the recovery mechanisms in this population in bigger samples is vital and deserves further investigation.

The FMS assesses stroke impairment and recovery in relation to the ability to perform arm movements in or out of synergy.^53^ Higher scores on the Fugl-Meyer indicate that individuals have the ability to move out of synergy. As such, it is a proxy of arm synergies however; further insights could be gained by measuring joint angles or muscle activation directly.^54^

## Conclusion

In a cohort of individuals with moderate hemiparesis, the strength of the CST, rather than the RST, determines initial and 12-week upper-limb impairment, as well as the capacity for skill-acquisition. In addition, the presence of abnormal synergies, as captured by the FMS, was not attributable to upregulation of the RST in either the deltoid or triceps muscles in the sub-acute stage. We therefore propose that synergy expression in the sub-acute period is related to an altered ratio of CST to RST activity rather than to an absolute change in RST connectivity strength.

## Supplemental Material

sj-pdf-1-nnr-10.1177_15459683211028243 – Supplemental Material for The Strength of the Corticospinal Tract Not the Reticulospinal Tract Determines Upper-Limb Impairment Level and Capacity for Skill-Acquisition in the Sub-Acute Post-Stroke Period Click here for additional data file. Supplemental Material, sj-pdf-1-nnr-10.1177_15459683211028243 for The Strength of the Corticospinal Tract Not the Reticulospinal Tract Determines Upper-Limb Impairment Level and Capacity for Skill-Acquisition in the Sub-Acute Post-Stroke Period by Ulrike Hammerbeck, Sarah F. Tyson, Prawin Samraj, Kristen Hollands, John W. Krakauer and John Rothwell in Neurorehabilitation and Neural Repair
