# Supplementary material for: The Strength of the Corticospinal Tract Not the Reticulospinal Tract Determines Upper-Limb Impairment Level and Capacity for Skill-Acquisition in the Sub-Acute Post-Stroke Period
Source: Neurorehabil Neural Repair. 2021 Jul 4;35(9):812–22. doi: 10.1177/15459683211028243 (PMC8414832; doi:10.1177/15459683211028243)
Supplement: sj-pdf-1-nnr-10.1177_15459683211028243 – Supplemental Material for The Strength of the Corticospinal Tract Not the Reticulospinal Tract Determines Upper-Limb Impairment Level and Capacity for Skill-Acquisition in the Sub-Acute Post-Stroke Period [file sj-pdf-1-nnr-10.1177_15459683211028243.pdf]

The strength of the corticospinal tract not the reticulospinal tract determines upper limb impairment level and capacity for skill acquisition in the sub-acute post-stroke period

Supplementary Information

Figure S1 The association between present or absent ipsilesional connectivity and impairment.

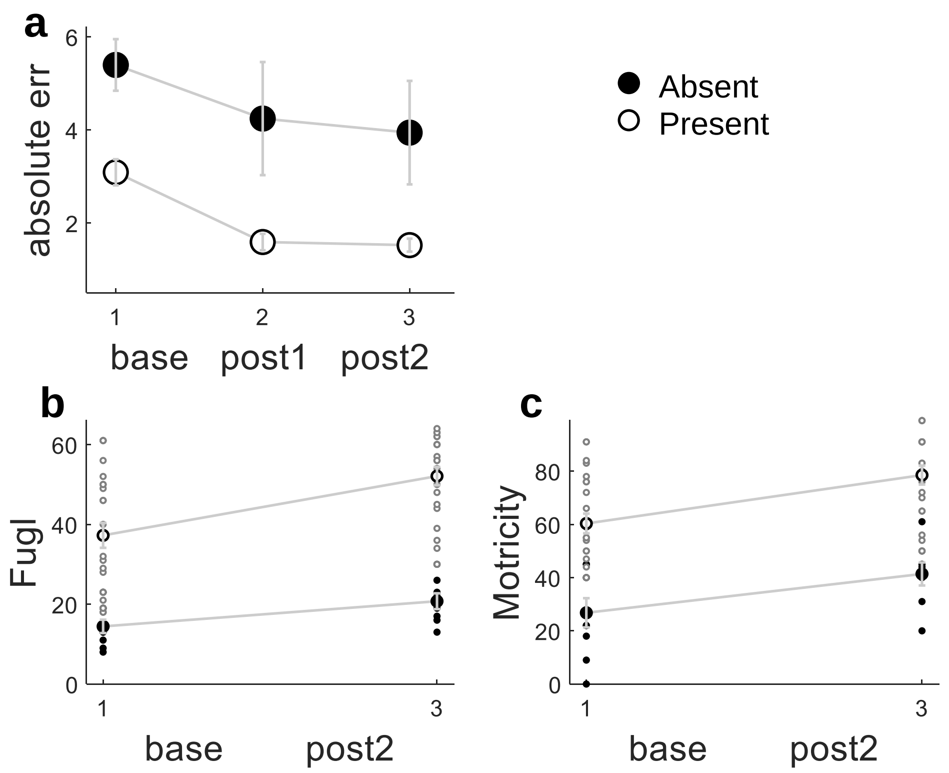

Reaching skill differed at baseline in individuals when categorised for either absent (n=17) or present (n=12) ipsilesional connectivity (Fig S1a) (main effect of connectivity $F_{(1,26)}$=2.9, p=0.098 Reaching skill improved over time (effect of Time $F_{(1,26)}$=26.74, p<0.001) without an interaction for connectivity. We also found an association between the presence of ipsilesional connectivity and the baseline FMS (Fig S1b)(main effect of connectivity $F_{(1,27)}$=25.5, p<0.001). The FMS changed over time without an interaction (effect of Time $F_{(1,27)}$=25.3 p<0.001). Similarly an association was observed for ipsilesional connectivity and the MI (Fig S1c) (main effect of Motricity $F_{(1,25)}$=30.5, p<0.001) which also changed over time (effect of Time $F_{(1,25)}$=25.0, p<0.001) without an interaction with connectivity.

Figure S2 The association between present or absent contralesional connectivity and impairment.

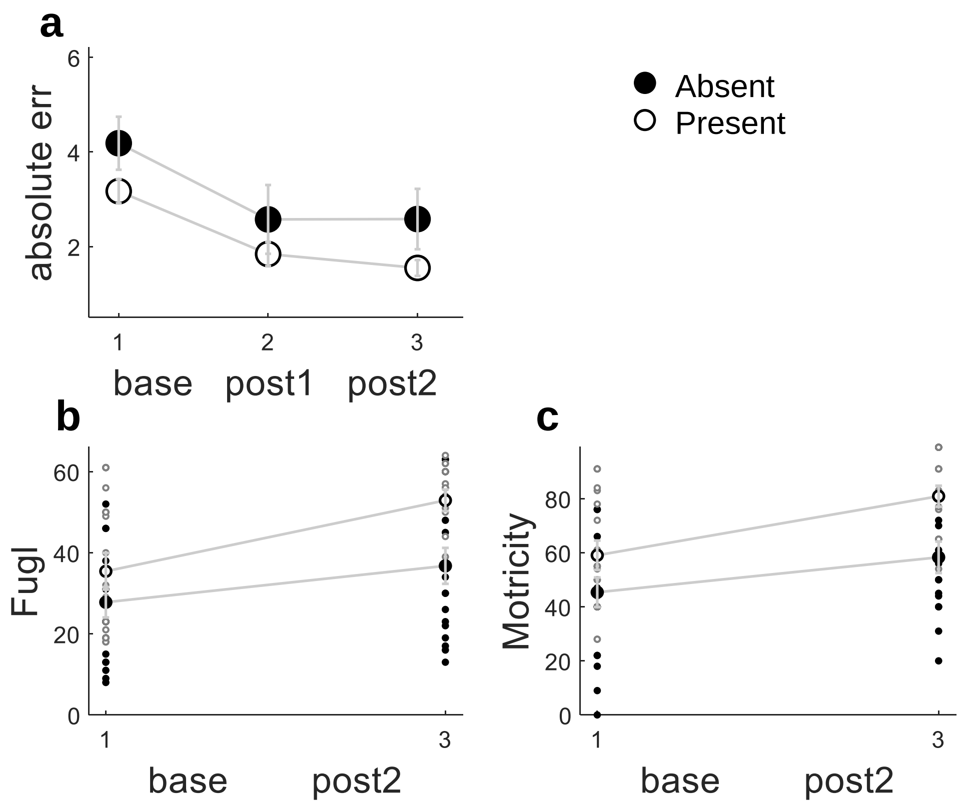

Reaching skill did not differ at baseline in individuals with absent(N=7) or present (n=22) contralesional connectivity (Fig S2 a) (main effect of connectivity $F_{(1,26)}$=2.9, p=0.098 Reaching skill improved over time (effect of Time $F_{(1,26)}$=26.74, p<0.001) without an interaction for connectivity.We found no association between the presence of contralesional connectivity and the FMS (Fig S2b)(main effect of connectivity $F_{(1,27)}$=2.8, p=0.106). The FMS changed over time without an interaction (effect of Time $F_{(1,27)}$=50.9, p<0.001). Neither was an association observed for the MI (Fig S2c) (main effect of Motricity $F_{(1,25)}$=1.3, p=0.268) which also changed over time (effect of Time $F_{(1,25)}$=45.0, p<0.001) without an interaction.
